# Rationally Designed Influenza Virus Vaccines That Are Antigenically Stable during Growth in Eggs

**DOI:** 10.1128/mBio.00669-17

**Published:** 2017-06-06

**Authors:** Alfred T. Harding, Brook E. Heaton, Rebekah E. Dumm, Nicholas S. Heaton

**Affiliations:** Department of Molecular Genetics and Microbiology, Duke University School of Medicine, Durham, North Carolina, USA; University of Pittsburgh School of Medicine

**Keywords:** antigenic instability, genetic engineering, influenza A virus, influenza B virus, vaccines

## Abstract

Influenza virus vaccine production is currently limited by the ability to grow circulating human strains in chicken eggs or in cell culture. To facilitate cost-effective growth, vaccine strains are serially passaged under production conditions, which frequently results in mutations of the major antigenic protein, the viral hemagglutinin (HA). Human vaccination with an antigenically drifted strain is known to contribute to poor vaccine efficacy. To address this problem, we developed a replication-competent influenza A virus (IAV) with an artificial genomic organization that allowed the incorporation of two independent and functional HA proteins with different growth requirements onto the same virion. Vaccination with these viruses induced protective immunity against both strains from which the HA proteins were derived, and the magnitude of the response was as high as or higher than vaccination with either of the monovalent parental strains alone. Dual-HA viruses also displayed remarkable antigenic stability; even when using an HA protein known to be highly unstable during growth in eggs, we observed high-titer virus amplification without a single adaptive mutation. Thus, the viral genomic design described in this work can be used to grow influenza virus vaccines to high titers without introducing antigenic mutations.

## IMPORTANCE

Influenza A virus (IAV) is a major public health threat, and vaccination is currently the best available strategy to prevent infection. While there have been many advances in influenza vaccine production, the fact that we cannot predict the growth characteristics of a given strain under vaccine production conditions *a priori* introduces fundamental uncertainty into the process. Clinically relevant IAV strains frequently grow poorly under vaccine conditions, and this poor growth can result in the delay of vaccine production or the exchange of the recommended strain for one with favorable growth properties. Even in strains that grow to high titers, adaptive mutations in the antigenic protein hemagglutinin (HA) that make it antigenically dissimilar to the circulating strain are common. The genomic restructuring of the influenza virus described in this work offers a solution to the problem of uncertain or unstable growth of IAV during vaccine production.

## INTRODUCTION

Influenza A virus (IAV), a member of the family *Orthomyxoviridae*, is a negative-sense RNA virus with a segmented genome (1). Seasonal IAV is a major public health concern, causing nearly 5 million cases of severe illness a year and an estimated 250,000 to 500,000 deaths (2). Vaccination is the main strategy used for limiting the public health burden of this virus, and neutralizing antibodies (Abs) directed against the hemagglutinin (HA) protein are thought to be the most important contributors to protection (3). Influenza virus vaccines are normalized based solely on HA content (4), and recombinant HA protein-only vaccines are FDA approved and currently in use (5).

Current trivalent and quadrivalent inactivated egg- and cell-based influenza vaccines rely on incorporating the glycoproteins from one of the desired strains into a standardized influenza virus genetic background, amplifying the virus, and then inactivating and partially purifying viral proteins for vaccination (6). The vaccine production process can be delayed due to poor growth of the reassortant viruses under laboratory conditions (7–9), and in extreme cases, the failure to grow a desired strain for vaccine production can lead to its complete exclusion from a multivalent vaccine formulation (10). This has been a problem particularly for recent human subtype H3 IAV strain-derived HA proteins that frequently display poor infectivity in embryonated chicken eggs (11–13).

Further, IAV vaccines are notorious for displaying variable rates of protection (14, 15). Poor vaccine efficacy is frequently blamed on improper vaccine strain selection or antigenic drift of circulating viruses; however, recent work has shown that the viral antigens acquire mutations during vaccine production, which leads to human vaccination with an antigenically dissimilar virus (16, 17). Thus, the ability to predictably grow any influenza virus strain to high titers, without altering the structure or antigenicity of the HA protein, would represent a significant improvement to current influenza virus vaccine production.

## RESULTS

To improve the vaccine production process, we decided to generate a replication-competent IAV that incorporated two different HA proteins onto the same virion. We reasoned that by pairing a laboratory-adapted HA with a second HA protein, derived from a circulating pathogenic IAV strain, we could ensure robust growth of the resultant IAV regardless of the growth characteristics of the second HA and thereby reduce the selective pressure on this HA to mutate.

To accomplish this goal, we needed to establish a genomic organization that would encode two functional HA proteins. There have been reports of successful exogenous expression of foreign reporter proteins from the polymerase segments as well as IAV segments 8 and 6 (reviewed in references 18 and 19). All of the previously published reports, however, either generated virus-reporter fusion proteins or left residual amino acids that would inactivate a second HA protein. Since all previously published strategies were unsuitable for our purposes, we first needed to develop new methods to insert proteins into IAV that would not result in modifications to these proteins. For a rapid readout of virus rescue, we began by attempting to insert fluorescent proteins.

**FIG 1:**
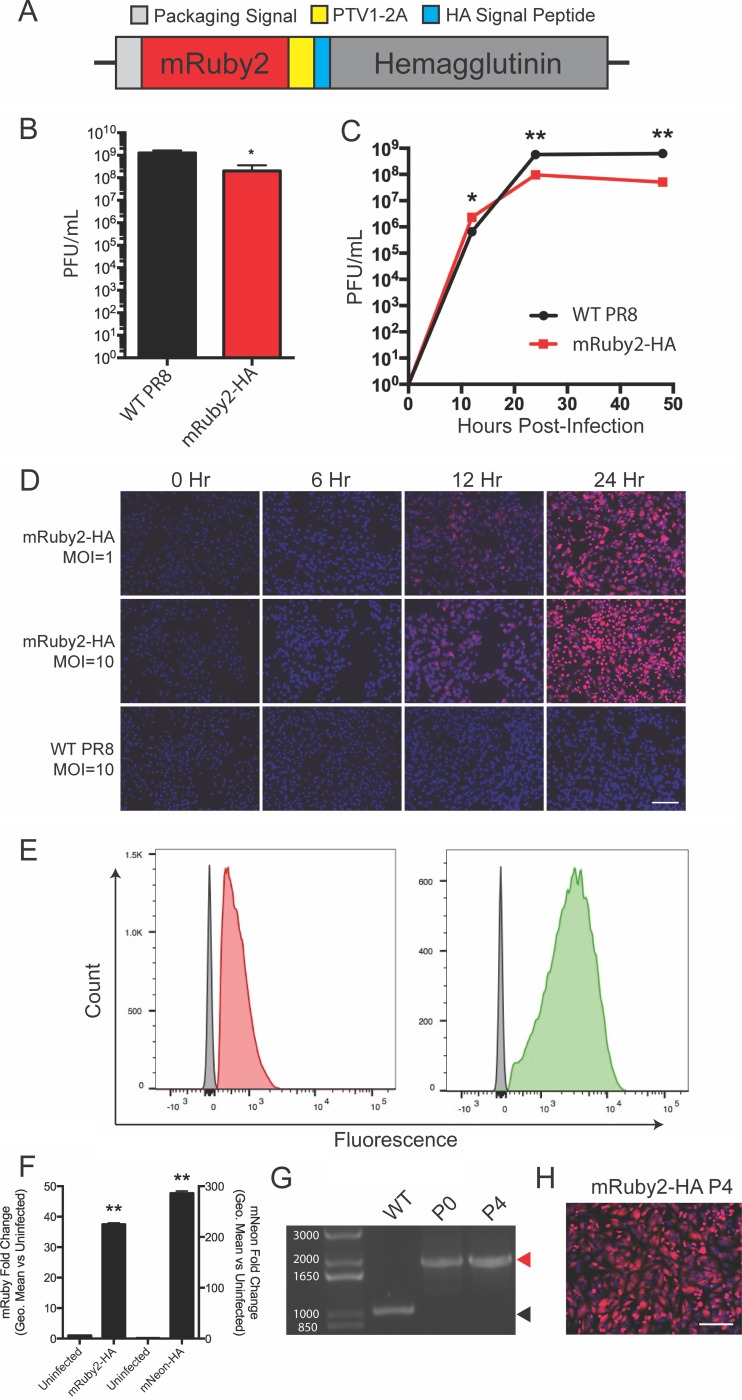
Encoding a red fluorescent reporter protein in segment 4 without leaving residual tags on the viral HA protein. (A) Diagram of the genomic segment 4 HA-based fluorescent reporter virus. (B) Endpoint titer of the mRuby2-HA virus compared to wild-type PR8 after 72-h incubation in eggs. (C) Multicycle growth kinetics of the mRuby2-HA virus on MDCK cells compared to WT. (D) Fluorescence microscopy time course of a single-cycle infection on MDCK cells comparing red fluorescence between wild-type and mRuby2-HA viruses. (E) Flow cytometry of mRuby2-HA- and mNeon-HA-infected cells (red or green, respectively) and uninfected cells (gray) represented as a histogram. (F) Quantification of the brightness of mRuby2-HA- and mNeon-HA-infected cells. (G) Viral segment 4 RT-PCR from wild-type PR8 and passages 0 and 4 of mRuby2-HA. The red arrowhead indicates the presence of the reporter gene; the black arrowhead indicates no reporter. The molecular weights of the DNA ladder bands are indicated in white. (H) Fluorescence microscopy of cells 24 h postinfection at an MOI of 1 with the passage 4 mRuby2-HA virus. *, *P* ≤ 0.05; **, *P* ≤ 0.001; bars, 100 μm (all panels).

Since the IAV HA gene encodes an N-terminal signal peptide which mediates appropriate subcellular localization and then is removed to generate the mature HA (20), we reasoned that encoding a fluorescent mRuby2 gene before the protein would not leave any additional amino acids on HA after signal peptide removal. To ensure that the signal peptide was recognized during translation, we engineered a porcine teschovirus 2A (PTV1-2A) motif to separate the fluorescent reporter and HA (Fig. 1A). Thus, the mRuby2 sequence should be released from the nascent polypeptide as the ribosome translates the PTV1-2A sequence and remain in the cytoplasm, while the HA signal peptide should be recognized and then removed during normal HA trafficking to the plasma membrane. We were able to rescue this virus in the H1N1 A/Puerto Rico/8/1934 (PR8) background and show that the resultant virus grew to high titers (Fig. 1B). In multicycle growth, the kinetics were similar to those of the parental strain (Fig. 1C). The HA segment is normally highly expressed in infected cells, and we were readily able to detect infected cells via microscopy and flow cytometry assays, with the brightness of the reporter related to the multiplicity of infection (MOI) (Fig. 1D and E). While red fluorescent proteins are useful to minimize signal overlap with green autofluorescence in tissue sections (21, 22), they display lower brightness than do green or yellow fluorescent proteins (23, 24). We therefore also rescued a virus expressing the exceptionally bright mNeonGreen protein (25) in the HA segment and performed flow cytometry (Fig. 1E and F). Quantification of the brightness of infected cells for both viruses was performed at 24 h postinfection, with an observed ~35-fold increase for the mRuby2 virus and an ~300-fold increase for the mNeon virus (Fig. 1F). Thus, we were able to encode foreign proteins in segment 4 of IAV with minimal effects on virus growth and no residual amino acids left on the viral HA protein. Finally, we assessed the stability of our mRuby2-HA virus over 4 serial passages in eggs. We observed no change in segment length or loss of fluorescence (Fig. 1G and H), indicating that the virus tolerates these manipulations. We also verified that the brightness of the virus did not decrease during the passaging, as well as performing plaque assays to ensure that no minor population of the stock had lost fluorescence (see Fig. S1A to C in the supplemental material).

10.1128/mBio.00669-17.1 FIG S1  The mRuby2-HA virus stably expresses the reporter protein over serial passaging. (A) Quantification of the brightness of passage 0 and passage 4 mRuby2-HA-infected cells via flow cytometry. (B) Table comparing the numbers of reporter-positive plaques out of total plaques between passage 0 and passage 4 of the mRuby2-HA virus. Identified plaques were confirmed via staining for influenza virus proteins as described in Materials and Methods. (C) Representative images of the plaques that were counted, demonstrating similar brightness and morphology between passages. Download FIG S1, PDF file, 0.5 MB. Copyright © 2017 Harding et al. 2017 Harding et al. This content is distributed under the terms of the Creative Commons Attribution 4.0 International license.

**FIG 2:**
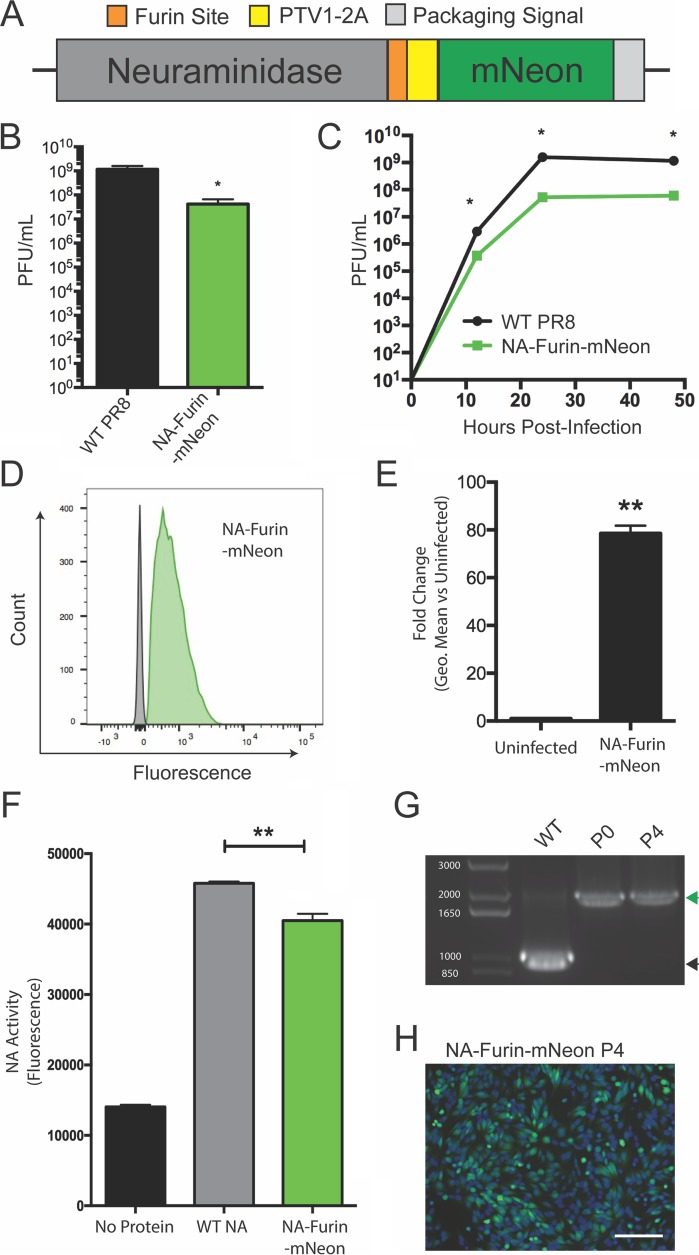
Encoding a green fluorescent reporter protein in segment 6 without leaving residual tags on the viral NA protein. (A) Diagram of the genomic segment 6 NA-based fluorescent reporter virus. (B) Endpoint titer of the NA-furin-mNeon virus compared to WT PR8 after 72-h incubation in eggs. (C) Multicycle growth kinetics of the NA-furin-mNeon virus on MDCK cells compared to WT. (D) Flow cytometry of NA-furin-mNeon-infected cells (green) and uninfected cells (gray) represented as a histogram. (E) Quantification of brightness of fluorescence in the NA-furin-mNeon-infected cells. (F) Comparison of neuraminidase activity of purified FLAG-tagged neuraminidase from WT PR8 and the NA-furin-mNeon virus. (G) Viral segment RT-PCR from wild-type PR8 and passages 0 and 4 of the NA-furin-mNeon virus. The green arrowhead indicates the presence of the reporter gene; the black arrowhead indicates no reporter. The molecular weights of the DNA ladder bands are indicated in white. (H) Fluorescence microscopy of cells 24 h postinfection at an MOI of 1 with the passage 4 NA-furin-mNeon virus. *, *P* ≤ 0.05; **, *P* ≤ 0.001; bars, 100 μm (all panels).

While our segment 4 design was successful in producing wild-type HA protein, there were residual C-terminal amino acids left from the PTV1-2A motif on the mRuby2 protein. Thus, we would ultimately not accomplish our goals by simply incorporating two HA proteins in segment 4. However, previous work has shown that foreign proteins can be expressed on the C terminus of the neuraminidase (NA) encoded in segment 6. Importantly, in those reports NA was forced to tolerate residual amino acids left on the C terminus after 2A-mediated protein separation (26, 27). To generate an untagged NA protein as a second, complementary approach to HA expression, we aimed to take advantage of cellular peptidases to remove tags left on the proteins at a PTV1-2A cleavage site. Previous work on recombinant protein expression has shown that after introduction of the furin cleavage site RKRR, recognition by furin protease and subsequent cleavage by carboxypeptidases completely eliminate the residual motif (28). We adapted this approach and encoded mNeon after the NA protein, separating the two proteins by a furin cleavage site and a PTV1-2A site (Fig. 2A).

Rescue and characterization of this virus showed that the virus grew to high titers and replicated with kinetics similar to those of the parental PR8 virus (Fig. 2B and C). Infected cells were readily detected, and the brightness was quantified via flow cytometry (Fig. 2D and E). To assess the activity of NA-furin-mNeon versus wild-type (WT) NA, we rescued viruses with FLAG-tagged versions of both neuraminidases (29) and then purified NA and performed a sialidase assay (Fig. 2F). The slight reduction in activity likely indicates that not all of the NA is fully processed by the furin protease as intended, potentially leaving some amino acids on the N terminus (diagrammed in Fig. S2). Finally, we assessed the stability of our NA-furin-mNeon virus over 4 serial passages. We again observed no loss of the reporter gene or decrease in brightness (Fig. 2G and H; Fig. S3A to C). Thus, we have developed two ways to express foreign proteins, one in segment 4 and one in segment 6, which leave little to no residual modification on the viral proteins and are well tolerated by the virus.

10.1128/mBio.00669-17.2 FIG S2  Schematic of the neuraminidase-furin-mNeon construct and its processing. (A) Depiction of the amino acids encoded by the construct. Amino acids are color coded to match the specific portions of the construct from which they come. (B) Depiction of the inability of ribosomes to form a peptide bond between the final glycine and proline of the PTV1-2A sequence, causing the neuraminidase and mNeon proteins to separate. (C) Depiction of furin protease recognizing the cleavage RKRR motif and cleaving the remaining PTV1-2A amino acids from neuraminidase. (D) Depiction of carboxypeptidase B enzymes cleaving the basic amino acids of the furin cleavage site from the N terminus of neuraminidase, leaving wild-type protein. Download FIG S2, PDF file, 0.2 MB. Copyright © 2017 Harding et al. 2017 Harding et al. This content is distributed under the terms of the Creative Commons Attribution 4.0 International license.

10.1128/mBio.00669-17.3 FIG S3  The NA-furin-mNeon virus stably expresses the reporter protein over serial passaging. (A) Quantification of the brightness of passage 0 and passage 4 NA-furin-mNeon-infected cells via flow cytometry. (B) Table comparing the numbers of reporter-positive plaques out of total plaques between passage 0 and passage 4 of the NA-furin-mNeon virus. Identified plaques were confirmed via staining for influenza virus proteins as described in Materials and Methods. (C) Representative images of the plaques that were counted, demonstrating similar brightness and morphology between passages. Download FIG S3, PDF file, 0.5 MB. Copyright © 2017 Harding et al. 2017 Harding et al. This content is distributed under the terms of the Creative Commons Attribution 4.0 International license.

**FIG 3:**
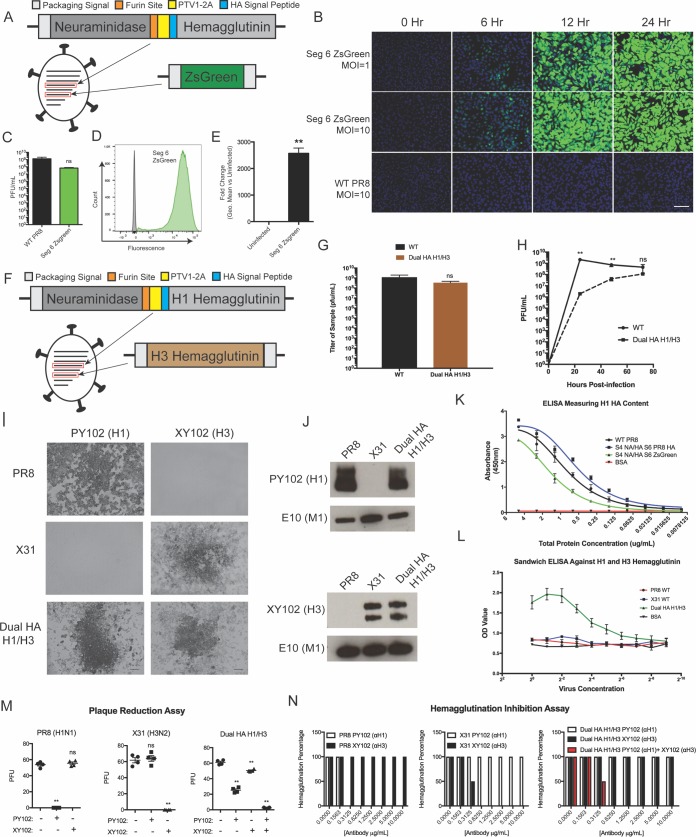
Expression of the HA and NA glycoproteins from a single segment allows the generation of a replication-competent H1/H3 dual-HA virus. (A) Diagram of the virus expressing both HA and NA glycoproteins in the genomic segment 4 and ZsGreen in the genomic segment 6. (B) Fluorescence microscopy time course of a single-cycle infection on MDCK cells comparing green fluorescence between wild-type and segment 4 NA/HA, segment 6 ZsGreen viruses. (C) Endpoint titer of the segment 4 NA/HA, segment 6 ZsGreen virus compared to wild-type PR8 after 72-h incubation in 10-day-old eggs. (D) Flow cytometry of the segment 4 NA/HA, segment 6 ZsGreen virus-infected (green) and uninfected (gray) cells represented as a histogram. (E) Quantification of the fold induction of fluorescence in infected cells over that in uninfected cells, caused by the segment 4 NA/HA, segment 6 ZsGreen virus. (F) Diagram of the H1/H3 dual-HA virus expressing both the subtype 1 HA and NA from genomic segment 4 and the subtype 3 HA from genomic segment 6. (G) Endpoint titer of the segment 4 NA/HA, segment 6 A/Hong Kong/1968 HA virus compared to wild-type PR8 after 72-h incubation in 11-day-old eggs. (H) Multicycle growth curve of the H1/H3 virus compared to wild-type PR8 after incubation in 11-day-old eggs. (I) Subtype-specific antibody staining of PR8, X31, and H1/H3 virus plaques. (J) Western blot assay of concentrated virus for the subtype 1 and 3 hemagglutinins. (K) ELISA measuring subtype 1 HA content utilizing a virus expressing two subtype 1 HAs from segment 4 and segment 6. (L) Sandwich ELISA of PR8, X31, and H1/H3 virus measuring content of H1 and H3 subtype HAs on the same virion. (M) Plaque reduction assays with subtype-specific H1 (0.1 μg/ml) and H3 (1 μg/ml) monoclonal antibodies. (N) Hemagglutination inhibition (HAI) assays utilizing antibodies to both subtype 1 and subtype 3 hemagglutinins. *, *P* ≤ 0.05; **, *P* ≤ 0.001; bars, 100 μm (all panels).

We theorized that by combining these strategies we could express both the HA and the NA glycoprotein from a single viral segment. We therefore encoded the NA protein, followed by a furin cleavage site, then PTV1-2A, and finally the HA protein, in segment 4 (Fig. 3A). Since seven-segment influenza viruses are known to grow poorly (30), we encoded the fluorescent protein ZsGreen in segment 6, where NA is normally encoded, as a placeholder (Fig. 3A). We successfully rescued this virus and observed that the virus expressed the reporter protein and grew to high titers (Fig. 3B and C) despite the reorganization of the glycoproteins. We also observed extremely high expression of the ZsGreen reporter protein (Fig. 3D and E), likely due to the addition of an artificial consensus Kozak signal in front of the reporter protein.

Since expressing HA and NA in a single segment was well tolerated by the virus, we returned to our original goal of a dual-HA IAV and designed a virus to express both a subtype 1 and a subtype 3 HA simultaneously (Fig. 3F). We encoded the original PR8 H1 protein in segment 4 (along with the NA protein) and encoded an additional H3 protein (from A/Hong Kong/1968) in segment 6 (where NA is normally located). We chose H1 and H3 HAs because these two IAV subtypes are currently circulating in humans, and we wanted to assay the ability of this technology to allow the incorporation of both subtype H1 and H3 HA proteins on the same virion. We were able to rescue this virus and found that it grew to high titers, with no statistical difference in endpoint titer from the parental PR8 strain, but with a delay in the kinetics of viral growth (Fig. 3G and H). We were also able to detect viral plaques with antibodies specific for either subtype 1 or subtype 3 HAs after infection with the H1/H3 dual-HA virus (Fig. 3I). The plaque size of the dual HA virus was reduced compared to the parental PR8 strain but was similar to the A/Hong Kong/1968-PR8 reassortant strain X31 (Fig. S4). We also determined the levels of the HA proteins in the dual-HA background relative to the H1 parent PR8 or the H3 parent X31 via Western blot analysis. Using purified virions, we observed that the H1/H3 virions packaged similar levels of the H1 and H3 glycoprotein to the single-HA parent strains (Fig. 3J).

10.1128/mBio.00669-17.4 FIG S4  Plaque morphology of the parental PR8 (H1N1) and X31 (H3N2) viruses compared to the dual-HA H1/H3 virus in a plaque assay on MDCK cells. Download FIG S4, PDF file, 1.3 MB. Copyright © 2017 Harding et al. 2017 Harding et al. This content is distributed under the terms of the Creative Commons Attribution 4.0 International license.

To quantify the total amount of HA on the surface of the virion, we rescued a double PR8 H1/H1 dual-HA virus and performed an enzyme-linked immunosorbent assay (ELISA) on the purified virions with an H1-specific antibody. We observed that the double-HA virus packaged more HA protein than the single HA parent, as expected from our Western blot analysis (Fig. 3K). NA activity levels were slightly reduced relative to parental PR8, likely indicating a slight reduction in the amount of the NA protein packaged (Fig. S5). We also performed a sandwich ELISA with monoclonal antibodies specific for the PR8 or HK68 HA to demonstrate that the two HA proteins were being packaged onto the same virion (Fig. 3L). In order to assay the stability of the second HA protein, we injected 20 embryonated chicken eggs with the dual H1/H3 virus. After 72 h of viral growth, a plaque assay was performed with each of the 20 viral populations. Plaques visible to the eye were stained with an H3-specific monoclonal antibody; we observed that every plaque was positive for the HK68 HA protein (Table S1).

10.1128/mBio.00669-17.5 FIG S5  Bivalent viruses have lower neuraminidase activity than WT PR8. Both WT PR8 and the dual-HA H1/H3 virus samples were concentrated and normalized to total protein. A sialidase activity assay was then performed, according to the standard procedures of the Sigma-Aldrich neuraminidase activity kit (MAK121), to evaluate NA content of each sample. Download FIG S5, PDF file, 0.3 MB. Copyright © 2017 Harding et al. 2017 Harding et al. This content is distributed under the terms of the Creative Commons Attribution 4.0 International license.

10.1128/mBio.00669-17.9 TABLE S1  Dual-HA A/Hong Kong/1/68-PR8 virus stably expresses the second HA in 20 independent parallel passages. Plaques that stained positive for A/Hong Kong/1/68 HA are shown out of the total plaques counted for each passage. Download TABLE S1, DOCX file, 0.1 MB. Copyright © 2017 Harding et al. 2017 Harding et al. This content is distributed under the terms of the Creative Commons Attribution 4.0 International license.

**FIG 4:**
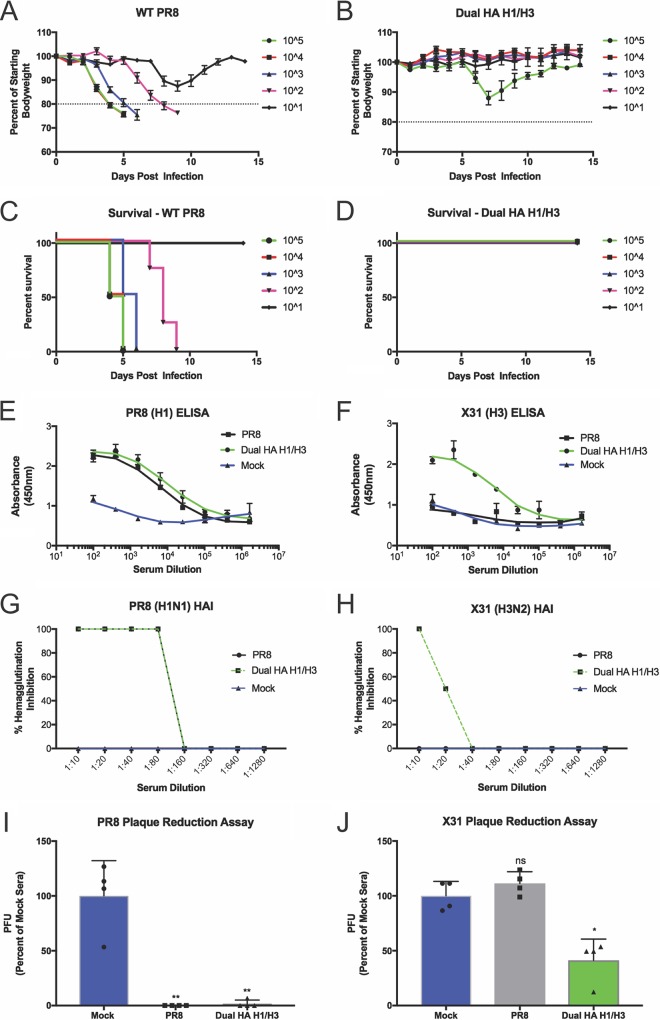
Infection with the live-attenuated H1/H3 dual-HA virus generates high levels of neutralizing antibodies to PR8 and X31. (A and B) Weight-loss curves from infections with the indicated doses of wild-type PR8 (A) or the H1/H3 dual-HA virus (B). (C and D) Survival curves from infections with the indicated doses of wild-type PR8 (C) or the H1/H3 virus (D). (E and F) H1-specific (E) or H3-specific (F) ELISA using sera from infected mice that received the highest dose of each strain and survived (PR8, 10^1^; H1/H3 dual HA, 10^5^). (G and H) HAI assays with subtype 1 (PR8) (G) and subtype 3 (X31) (H) HA viruses with pooled RDE-treated sera from mice that received the highest dose of infection and survived (PR8, 10^1^; H1/H3 dual HA, 10^5^). (I and J) Plaque reduction assays with PR8 (I) or X31 (J) using pooled RDE-treated sera from infected mice diluted 1:25. *, *P* ≤ 0.05; **, *P* ≤ 0.001 (all panels).

We next tested the functionality of both the H1 and H3 HAs in our dual-HA virus. We incubated our dual-HA virus with neutralizing monoclonal antibodies specific for either the H1 or the H3 HA. We observed that only when we mixed the two antibodies together were we able to completely neutralize the dual H1/H3 virus (Fig. 3M and N). We reasoned that the delay in viral replication kinetics due to increased genome size would significantly attenuate the virus. We therefore tested the ability of the H1/H3 virus to act as a live attenuated vaccine without additional mutations. C57BL/6 mice were infected with a range of doses of either the parental PR8 strain or the H1/H3 strain. Despite high morbidity and mortality of the parental PR8 strain, our H1/H3 virus caused no mortality at the tested doses (Fig. 4A to D). Despite the difference in disease, high levels of antibodies were elicited by the H1/H3 virus infection in surviving animals (Fig. 4E and F). Furthermore, these antibodies were found to neutralize virus at similar levels as those elicited from the parental PR8 infection, as determined by HA inhibition (HAI) and plaque reduction assays (Fig. 4G to J). Sera used for both HAI and plaque reduction assays were treated with sialic acid receptor-destroying enzyme (RDE) to eliminate nonspecific inhibition of viral binding mediated by serum components other than antibodies (31, 32).

**FIG 5:**
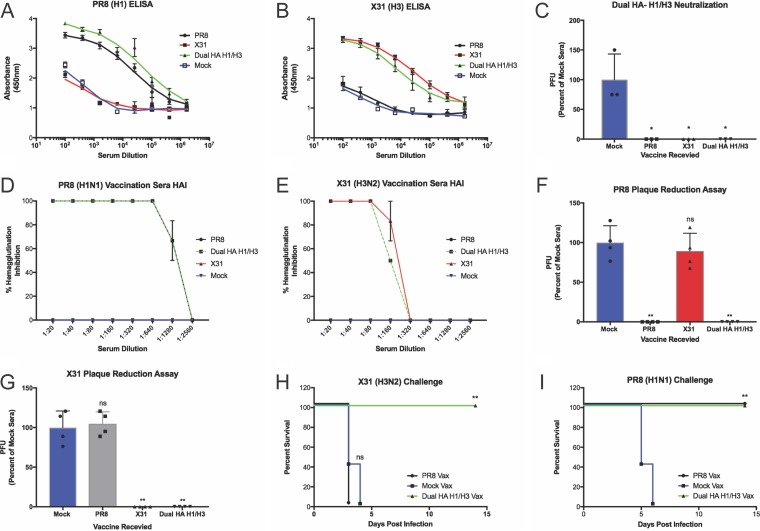
Vaccination with inactivated H1/H3 dual-HA virus generates high levels of protective antibodies to PR8 and X31. (A and B) H1-specific (A) or H3-specific (B) ELISA from vaccinated mouse serum. (C) Neutralization of the H1/H3 dual-HA influenza virus with polyclonal mouse sera raised against H1-, H3-, or H1/H3-expressing viruses. (D and E) HAI assays with subtype 1 (PR8) (D) and subtype 3 (X31) (E) HA viruses with pooled RDE-treated sera from vaccinated mice. (F and G) Plaque reduction assays with PR8 (F) or X31 (G) using pooled RDE-treated sera from vaccinated mice diluted 1:25. (H and I) Challenge experiments with X31 (H) or PR8 (I) in mice receiving inactivated PR8 or H1/H3 dual-HA virus vaccination. *, *P* ≤ 0.05; **, *P* ≤ 0.001 (all panels).

Since most IAV vaccines are inactivated, we also wanted to evaluate the dual-HA virus in this context. To inactivate the virus for administration, we formalin treated either the H1N1 (PR8), H3N2 (X31), or H1/H3 dual-HA virus and intramuscularly vaccinated mice. After vaccination and a single boost, we found that mice vaccinated with either PR8 or X31 produced high levels of the corresponding HA antibodies (Fig. 5A and B). Mice vaccinated with PR8 and X31, however, showed no detectable antibody response to the reciprocal HA, while the dual-HA H1/H3 virus vaccination led to equal or higher levels of antibodies to both of the HAs relative to the single-HA vaccines.

We next wanted to characterize the safety profile of our dual-HA virus in more detail. We therefore incubated the H1/H3 virus with polyclonal sera reactive against either PR8 or X31. Both of these sera were able to completely neutralize the dual-HA virus, showing that preexisting immunity from vaccination to one of the HA subtypes is sufficient to neutralize a “bivalent” virus (Fig. 5C). HAI and plaque reduction assays with RDE-treated sera raised against the H1/H3 virus revealed functional inhibition of PR8 and X31 receptor binding and virus infection, respectively, with the same efficacy as sera derived from vaccination with either of the single-HA parents alone (Fig. 5D to G; Fig. S6). Finally, we performed challenge experiments to show protection after vaccination *in vivo*. While vaccination with PR8 was able to protect from PR8 challenge, it was unable to protect from X31 challenge (Fig. 5H and I; Fig. S7A and B). Similarly, vaccination with the monovalent X31 virus was able to protect against X31 challenge, but it was not able to protect against PR8 challenge (Fig. S7C to F). Vaccination with the H1/H3 dual-HA virus, however, fully protected mice from challenge with either PR8 or X31 (Fig. 5H and I; Fig. S7A and B), indicating that the antibodies generated after H1/H3 virus vaccination are protective.

10.1128/mBio.00669-17.6 FIG S6  Plaque reduction assays performed with sera from vaccinated mice. Plaque reduction assays were repeated at a dilution two times higher (1:50) than that reported in Fig. 5F and G against the PR8 (H1N1) virus (A) and X31 (H3N2) virus (B). Download FIG S6, PDF file, 0.2 MB. Copyright © 2017 Harding et al. 2017 Harding et al. This content is distributed under the terms of the Creative Commons Attribution 4.0 International license.

10.1128/mBio.00669-17.7 FIG S7  Weight loss and survival curves from X31 and PR8 challenges of vaccinated mice. Mice were vaccinated with 7 μg of protein from concentrated samples of either inactivated PR8 WT, X31, or bivalent virus. After 2 weeks, mice were boosted and then challenged with the H3N2 strain X31 (A, C, and E) or the H1N1 strain PR8 (B, D, and F). Each cage of mice (*n* ≥ 4) was weighed daily for 14 days, and the average percent weight loss was recorded. Download FIG S7, PDF file, 0.9 MB. Copyright © 2017 Harding et al. 2017 Harding et al. This content is distributed under the terms of the Creative Commons Attribution 4.0 International license.

**FIG 6:**
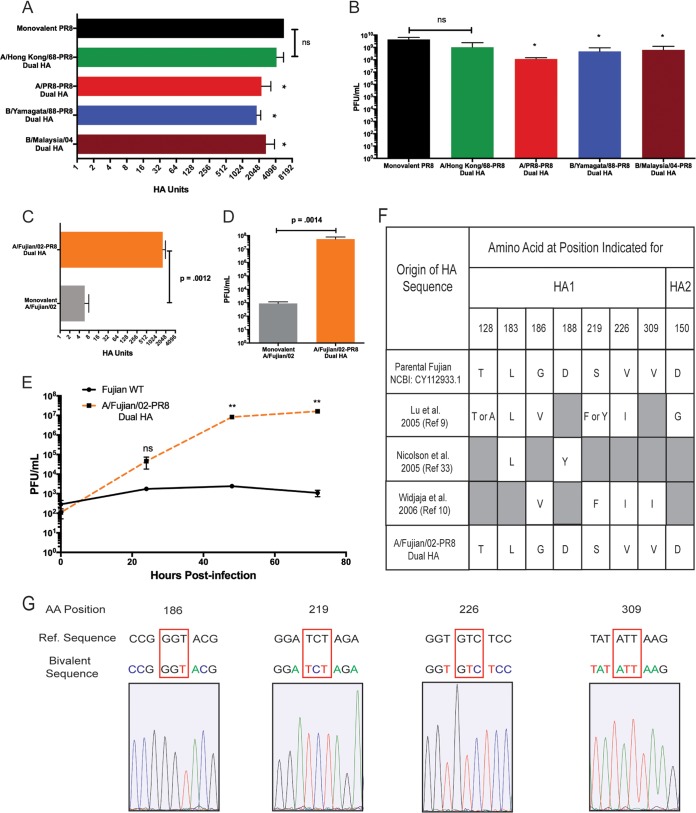
Dual-HA viruses can be generated with a variety of HA proteins and are antigenically stable during growth in eggs. (A) Hemagglutination units of the indicated dual-HA viruses from various IAV and IBV strains (H3, A/Hong Kong/1968; H1, A/Puerto Rico/8/1934; B Yamagata lineage, B/Yamagata/1988; B Victoria lineage, B/Malaysia/2004) relative to the parental PR8 strain. (B) Titers of the viruses from panel A. (C) Hemagglutination units of a dual-HA virus expressing the A/Fujian/411/2002 HA relative to the mono-HA A/Fujian/411/2002 WT. (D) Endpoint titers of the viruses from panel C. (E) Multicycle growth comparing the 6+2 reassortant in the PR8 background with A/Fujian/411/2002 glycoproteins and the dual-HA A/Fujian/411/2002-PR8 viruses. (F) Comparison of the parental A/Fujian/411/2002 sequence with the dual-HA virus after growth in eggs, along with previously published reports of mutations that occur in the HA of A/Fujian/411/2002 which are required to allow egg growth. (G) Sequencing chromatograms of the A/Fujian/411/2002 HA in the bivalent background after egg growth. Red boxes indicate positions that have been previously published to mutate upon egg adaptation. *, *P* ≤ 0.05; **, *P* ≤ 0.001; ns, not significant (all panels).

We next evaluated the breadth of HA proteins that could be expressed in the context of a double-HA virus. Current quadrivalent IAV vaccines are a mixture of IAVs with a subtype 1 and subtype 3 HA, as well as influenza B viruses from both the Victoria and Yamagata lineages. We therefore rescued double-HA viruses with the PR8 HA and a representative HA from each of these strains. We observed robust growth (without any HA mutations) for all of the recombinant viruses (Fig. 6A and B), indicating that there was no functional interference between the two HA proteins. To test our approach with a current and clinically relevant H3 strain, we rescued a dual-HA virus expressing the HA from the A/Victoria/210/2009 strain, which was included in the Fluarix quadrivalent vaccine produced by GlaxoSmithKline for 2017 to 2018. As expected, this virus grew to levels similar to those of our other bivalent viruses, and upon sequencing after several rounds of growth in eggs, we detected no mutations in the entire open reading frame (ORF) of the A/Victoria/210/2009 HA (Fig. S8).

10.1128/mBio.00669-17.8 FIG S8  Dual-HA viruses using modern H3 HA exhibit similar growth kinetics and HA content as egg-adapted dual-HA viruses. (A) HA assay of A/Victoria/210/09 virus expressing dual HA compared to A/Hong Kong/1968 dual HA. (B) Growth kinetics in 11-day-old eggs of A/Victoria/210/2009 virus expressing dual HA compared to the A/Hong Kong/1968 dual-HA virus. Download FIG S8, PDF file, 0.6 MB. Copyright © 2017 Harding et al. 2017 Harding et al. This content is distributed under the terms of the Creative Commons Attribution 4.0 International license.

Finally, we wanted to evaluate the antigenic stability of an HA protein that is normally unstable during growth in embryonated chicken eggs. We selected the HA from A/Fujian/411/2002 (Fujian02), which is a well-characterized H3N2 strain that is known to grow extremely poorly in eggs and rapidly acquires adaptive HA mutations to facilitate growth (9, 10, 33). After rescuing a dual PR8/Fujian02 HA virus and growing a purified stock (which represents several rounds of growth), we observed high-titer growth in embryonated chicken eggs that was significantly increased (~4 orders of magnitude) compared to the standard 6+2 reassortant in the PR8 background (Fig. 6C to E). Previous work has shown that robust growth of A/Fujian/411/2002 requires the acquisition of several characteristic mutations to the HA protein (9, 10, 33). We therefore extracted RNA from our egg-grown dual PR8/Fujian02 population and sequenced the A/Fujian/411/2002 HA encoded in segment 6. Sequence analysis confirmed that the HA sequence of our dual-HA virus did not contain any of the previously identified adaptive mutations (Fig. 6F and G). In fact, there was not a single adaptive mutation in the entire ORF of the A/Fujian/411/2002 HA.

## DISCUSSION

We began this study with the goal of developing a viral genetic system that would allow antigenically stable, high-titer growth of influenza viruses for vaccine production regardless of the intrinsic properties of a specific HA protein. We accomplished this task by expressing two functional HA proteins on a single, replication-competent virus. We first developed new methods to express untagged proteins from IAV segments 4 and 6 and subsequently combined the HA and NA proteins into segment 4. The final step was to add an additional HA into segment 6. Previous attempts to make bivalent influenza viruses have had limited success; the viruses required extensive genome alterations, which resulted in a major decrease in fitness—greater than a 3-log_10_ reduction in titer (34, 35). Our dual-HA viruses grew to high titers (~10^8^ PFU/ml) and in some cases grew to titers indistinguishable from the parental PR8 strain (~10^9^ PFU/ml). Our approach required modifications to only segments 4 and 6. Thus, this technology is fully compatible with current vaccine production methods which insert segments 4 and 6 from a circulating strain into a standardized genetic background (36). Importantly, our dual-HA viruses displayed fundamentally reduced virulence, almost certainly due to the drastically increased genome size and the resulting effects on viral replication kinetics. The virus is also completely neutralized by polyclonal sera raised against either of the hemagglutinins, further highlighting the safety of this design.

Our dual-HA virus approach was designed to promote recombinant virus growth irrespective of the nature of the specific HA used and to fully preserve the viral antigenic epitopes. Some human strains of influenza virus (especially H3N2 strains) initially grow poorly as reassortants in embryonated chicken eggs (11, 37), which delays vaccine production. Recently, in 2009, poor growth of pandemic A/California/07/2009 H1N1 swine flu vaccine candidates delayed vaccine production by months (7, 8), and in 2002, the H3N2 A/Fujian/411/2002 strain grew so poorly that although it was the major circulating strain at the time, it could not be included in the seasonal vaccine (9, 10). This led to vaccine/circulating strain mismatch and poor vaccine efficacy in 2003 and 2004 (38). We have directly demonstrated the utility of our approach by generating a dual-HA version of the A/Fujian/411/2002 strain. We observed immediate, robust virus growth in chicken eggs that was substantially higher than that with a standard reassortant of A/Fujian/411/2002, and there was no requirement for adaptive mutations. The implication of our data is that, had this technology been available in 2002, A/Fujian/411/2002 could have been grown and included in the seasonal vaccine and human influenza that season likely would have been significantly reduced.

The other major goal of this study was to preserve the antigenicity of a human HA protein during growth in embryonated chicken eggs, where variants with altered antigenicity frequently arise due to differences in virus receptor structures between mammals and birds (39–41). Studies have shown that even in years when the strain selected for vaccine production matches the circulating strain, mutations acquired during amplification of the vaccine strain can lead to poor protection after vaccination (16, 42). Our results showed that by pairing an HA that allows high-titer growth under the growth conditions of interest (such as that from PR8) with a clinically relevant HA that is known to mutate easily (such as that from A/Fujian/411/2002), the selective pressure to fix adaptive mutations in the second HA can be entirely eliminated. While difficult-to-grow strains can eventually be adapted to grow to high titers in eggs, this requires the serial passage of the relevant IAV reassortant in eggs, resulting in the acquisition of adaptive mutations in the viral glycoproteins, which must then be carefully screened for effects on antigenicity (37). Our dual-HA genetic approach completely eliminated the need for this time-consuming step.

We also observed increased HA density on the surface of the dual-HA virion, which by definition increases the amount of HA antigen relative to other viral proteins. Thus, a dual-HA virus has the potential to deliver the same amount of HA antigen in a smaller amount of total protein, which may increase vaccine tolerance and decrease side effects. Finally, this technology is not restricted to expressing solely influenza virus proteins. It can theoretically also be used as a platform to produce vaccines with a combination of influenza virus and non-influenza virus antigens while nevertheless still utilizing the current influenza vaccine production infrastructure.

In conclusion, we have developed two independent ways to express foreign proteins in IAV and combined those approaches to generate a replication-competent, dual-HA “bivalent” virus. We have shown that our viruses require no adaptation step and allow high-titer, antigenically stable growth of essentially any clinically relevant influenza A or B virus HA protein. This technology is fully compatible with current vaccine production practices and can be immediately utilized to facilitate rapid and cost-effective production, as well as to potentially increase the protective efficacy, of influenza virus vaccines.

## MATERIALS AND METHODS

### Cells and antibodies.

Madin-Darby canine kidney (MDCK) cells were grown in minimal essential medium (MEM) supplemented with 10% fetal bovine serum, HEPES, NaHCO_3_, GlutaMAX, and penicillin-streptomycin. 293T cells were grown in Dulbecco’s modified Eagle’s medium (DMEM) supplemented with 10% fetal bovine serum, GlutaMAX, and penicillin-streptomycin. Monoclonal antibodies specific for PR8 H1 (PY102), HK68 H3 (XY102), and influenza virus M1 (E10) were provided by Tom Moran at the Experimental Therapeutics Institute at the Icahn School of Medicine at Mount Sinai.

### Cloning and rescue of recombinant viruses.

Recombinant viruses were generated as previously described (43) by use of the bicistronic pDZ rescue plasmid system. Viral protein sequences were generated from rescue plasmids from the A/Puerto Rico/8/1934 H1N1 background. Fluorescent proteins and linker sequences were synthesized using influenza A virus codon usage preferences (IDT), and viral packaging signals were used as previously described (44). Primer sequences are listed in Table S2 in the supplemental material. The PR8 NA FLAG virus has been previously described (29). InfusionHD (Clontech) or the NEBuilder HiFI DNA assembly kit (NEB) was used to assemble DNA fragments before transformation into Clontech Stellar competent cells, per the manufacturer’s instructions. Insert size was then confirmed by colony PCR, and inserts were sequenced via Sanger sequencing before use in viral rescue. The plasmid (kindly provided by Richard Webby) for the rescue of the WT A/Fujian/411/2002 reassortant virus has the same sequence as that deposited in GenBank under accession number CY112933.1 with three nonsynonymous nucleotide changes, a T to A at position 610 and an A to G at positions 736 and 987. The following viral HA genes were synthesized (IDT) with silent mutations to eliminate the normal packaging signals. The sequence used for the A/Fujian/411/2002 dual-HA virus is the same as that deposited in GenBank under accession number CY112933.1 with one nonsynonymous nucleotide change, a G to T at position 769. The sequence used for the A/Victoria/210/2009 dual-HA virus is the same as that deposited in GenBank under accession number HM459583.1. The sequence used for the B/Malaysia/2004 dual-HA virus is the same as that deposited in GenBank under accession number CY119706.1 with two nonsynonymous nucleotide changes: an A to G at position 42 and a C to T at position 638. The sequence used for the B/Yamagata/1988 dual-HA virus is the same as that deposited in GenBank under accession number CY018765.1 with three nonsynonymous nucleotide changes, a G to A at positions 484 and 645 and a C to A at position 652. Virus rescue plasmids were transfected into 293T cells using the Mirus TransIT-LT1 reagent along with the remaining viral RNA segments from WT PR8. Rescued virus was then propagated in 10-day-old chicken eggs (Charles River) at 37°C for 72 h.

10.1128/mBio.00669-17.10 TABLE S2  Primers used in this study (all sequences shown 5′ to 3′). Download TABLE S2, DOCX file, 0.1 MB. Copyright © 2017 Harding et al. 2017 Harding et al. This content is distributed under the terms of the Creative Commons Attribution 4.0 International license.

### Viral titer.

Prior to titer determination, 30 to 50 PFU of each dilution-purified stock of virus was injected into eggs and incubated at 37°C for 72 h. Then, the allantoic fluid was collected, and viral titer was determined via standard plaque assay procedures on MDCK cells. Cells were incubated for 1 h in 333 μl of diluted virus suspension at 37°C, before removing the virus and applying the agar overlay. Cells were then incubated at 37°C for 72 h before being fixed in 4% paraformaldehyde (PFA) in phosphate-buffered saline (PBS) for at least 4 h. The 4% PFA was then aspirated, and the agar layer was removed before washing cells in PBS and incubating them at 4°C overnight in mouse serum from PR8-infected mice. Mouse serum was diluted 1:2,000 in antibody dilution buffer, which was made using 5% (wt/vol) nonfat dried milk and 0.05% Tween 20 in PBS. After the overnight incubation in primary antibody, plaque assays were washed with PBS three times and then incubated for 1 h in anti-mouse IgG horseradish peroxidase (HRP)-conjugated sheep antibody (GE Healthcare) diluted 1:4,000 in antibody dilution buffer. Assays were then washed three additional times with PBS and incubated in 0.5 ml of True Blue reagent for 30 min to allow for the staining of plaques. Once plaques were visible, plates were washed with water and allowed to dry before counting (only wells with greater than 5 plaques were used for the calculation of endpoint titer).

### Microscopy time course.

Microscopy images were taken using MDCK cells infected with various MOIs of either the reporter virus or WT virus. Cells were infected in 300 μl of virus for 1 h at 37°C; after this incubation period, the infection medium was removed and cells were placed in complete medium. At the indicated time after infection, MDCK cell medium was removed and replaced with 1 ml of warm PBS. Cells were then incubated with Hoechst stain (0.5 μl/ml of PBS) to allow for the staining of nuclei, and imaging was performed on the Zoe fluorescent cell imager (Bio-Rad). Images were then processed with ImageJ (NIH).

### Flow cytometry.

MDCK cells were infected for approximately 24 h before being trypsinized and collected for flow cytometry. Raw data were collected on a FACSCanto II (BD) machine, and data were processed with FlowJo software.

### Viral passaging and RT-PCR.

Virus was passaged in 10-day-old eggs purchased from Charles River Laboratories, Inc. Thirty to 50 PFU of each virus was injected into two eggs for each passage. Eggs were incubated for 72 h at 37°C in a humidified egg incubator before collection of the allantoic fluid. Virus was confirmed in the sample by hemagglutination assay before being injected into a new set of eggs. The passage 0 and 4 samples were subjected to Trizol RNA extraction. Reverse transcription-PCR (RT-PCR) was performed using the Superscript III one-step RT-PCR kit according to the manufacturer’s instruction with segment-specific primers. Samples were run on a 1% agarose gel and imaged. Microscopy images were taken 24 h postinfection, and microscopy was performed in the same manner as that described previously for time courses.

### Western blotting.

Virions were concentrated using a 30% sucrose cushion for 1 h at 25,700 rpm on the Sorvall TH-641 swinging bucket rotor. Equal amounts of protein were loaded into 4 to 20% acrylamide gels and transferred to a nitrocellulose membrane. Five percent nonfat dry milk in PBS plus 0.1% Tween 20 was used to block for 1 h, and a 1:1,000 dilution of primary antibody PY102, XY102, or E10 was incubated overnight. An anti-mouse–HRP secondary antibody was incubated for 1 h, and the blot was exposed to film. The membrane was then stripped for reprobing with the E10 M1 antibody.

### HAI assay.

Hemagglutination inhibition (HAI) assays were performed using 10^7^ PFU of virus per well, diluted in cold PBS. These samples were then mixed with a range of dilutions of monoclonal antibodies or sera collected from vaccinated mice. All data shown containing sera are from pooled, receptor-destroying enzyme (RDE)-treated samples. All samples were treated according to Denka Seiken Co.’s protocol with RDE (II) Seiken (catalog no. 370013). Once virus and antibody were mixed together, an equal amount of chicken blood diluted 1:40 in cold PBS was mixed with each sample and incubated at 4°C for approximately 30 min.

### Plaque reduction assay.

All plaque reduction assays were performed on MDCK cells. Virus was diluted to 50 PFU and mixed with antibody before being incubated at room temperature for 30 min. The virus-antibody mixture was then applied to the cells and incubated for an additional 30 min at 37°C, shaking the samples every 10 to 15 min to ensure that cells were evenly covered by the mixture. After the incubation, the solution was aspirated and an agar overlay was applied. Plaque assays were then performed as described above, and plaques were counted. All data shown containing sera are from pooled, receptor-destroying enzyme (RDE)-treated samples. All samples were treated according to Denka Seiken Co.’s protocol with RDE (II) Seiken (catalog no. 370013).

### Sandwich ELISAs.

For the sandwich enzyme-linked immunosorbent assay (ELISA), 96-well plates were coated with 100 μl of 5 μg/ml of mouse anti-H3 XY102 (IgG2) by overnight incubation at 4°C in a carbonate buffer. Plates were then washed twice with 150 μl of PBS and blocked with 1% bovine serum albumin (BSA) in PBS for 1 to 2 h at room temperature. A 2-fold serial dilution in the blocking buffer was then added to the plate and incubated overnight at 4°C (a starting concentration of 5% BSA was used for the BSA control). After this incubation, plates were washed twice with PBS and then incubated with 100 μl of 1 μg/ml of the subtype H1-specific antibody PY102 (IgG1) for 3 h at 37°C, and the antibody was detected by goat anti-mouse IgG1 conjugated with HRP (Thermo Fisher Scientific) (1:2,000).

### ELISAs.

Virions were concentrated using a 30% sucrose cushion for 1 h at 25,700 rpm on the Sorvall TH-641 swinging bucket rotor. Samples were then resuspended in 1 ml of PBS, and protein concentration was determined via the Bradford method. Ninety-six-well plates were then coated at 4°C with a range of protein concentrations using a carbonate buffer overnight. All samples were diluted to the same starting concentration (5% BSA was used as the starting concentration for the BSA control). Plates were then washed twice with 150 μl of PBS and blocked with 1% BSA in PBS for 1 to 2 h at room temperature. After this incubation, plates were washed twice with PBS and then incubated overnight at 4°C in 100 μl of a mixture of 1:2,000 PY102 (an H1-specific mouse antibody) and 1:1,000 XY102 (an H3-specific mouse antibody) diluted in 1% BSA-PBS. Plates were then washed twice with PBS and incubated for 1 to 2 h at room temperature in 100 μl of 1% BSA-PBS containing 1:5,000 goat anti-mouse HRP-conjugated antibody (Ab). Plates were then washed twice with PBS and incubated in tetramethylbenzidine (TMB) HRP substrate for approximately 20 min. At this time, or when the lowest dilution began to saturate with color, 100 μl of 1 M sulfuric acid was added to each well to stop the reaction and absorbance was measured at 450 nm on a plate reader.

### Animal infections.

Eight- to 10-week-old C57BL/6 mice were used for all experiments, with a sample size of at least 4 mice per dose of virus. Prior to infection, mice were anesthetized with a 100-μl injection of a ketamine-xylazine mixture. Mice were weighed and marked, and 40 μl of virus diluted in pharmaceutical-grade PBS was administered intranasally. Mice were weighed daily and euthanized once their body weight reached 80% of the starting weight measured prior to infection as a humane endpoint. Euthanasia was performed via CO_2_ as the primary method, and a bilateral thoracotomy was performed as the secondary method. Viral challenge of vaccinated mice was performed using this procedure as well. All procedures were approved by the Duke University IACUC.

### Vaccination of mice.

Mice were vaccinated with inactivated virus in order to examine the potential efficacy of our virus as a vaccine. Virus was concentrated and inactivated with PFA. Prior to injection, PFA was removed with Thermo Scientific Slide-a-Lyzer dialysis cassettes according to manufacturer instructions. Protein samples were then diluted to 70 μg/ml in pharmaceutical-grade PBS. Mice were sedated as previously mentioned, and a 100-μl vaccination was administered intramuscularly into the right leg of each mouse. After 2 weeks, mice were vaccinated once more in the same fashion and given another 2-week period before challenge or collection of serum.

### Cell-based ELISA.

293T cells were trypsinized and resuspended in 293T medium at a concentration of 1 × 10^5^ cells/ml and plated on 96-well plates that were poly-l-lysine treated. A transfection mixture was made with 900 μl of Opti-MEM, 30 μl of TransIT-LT1, and 10 μg of DNA (either PR8 [H1] hemagglutinin in the pDZ plasmid or HK68 [H3] hemagglutinin in the pDZ plasmid). This mixture was incubated for 5 min before being added to the 293T cells in suspension. Plates were incubated at 37°C for 2 days before fixation in 100 μl of 4% PFA for 5 min. Plates were then put through the same ELISA procedure listed above.

### Neuraminidase activity assay.

FLAG-tagged neuraminidase from both WT PR8 and the NA-furin-mNeon virus was concentrated and purified from virions using Sigma-Aldrich anti-FLAG M2 magnetic beads (M8823) according to the manufacturer’s protocol. A Bradford assay was then performed to measure protein concentration and standardize the samples. Once this was done, the Sigma-Aldrich neuraminidase activity assay kit (MAK121) was used according to the manufacturer’s protocol to evaluate the activity of the respective neuraminidase proteins.

### Statistical analysis.

Comparison of data sets was performed using an unpaired, two-tailed Student *t* test unless otherwise stated. Analysis was performed using Prism 7 (GraphPad) software.
