# Supplementary material for: Rationally Designed Influenza Virus Vaccines That Are Antigenically Stable during Growth in Eggs
Source: mBio. 2017 Jun 6;8(3):e00669-17. doi: 10.1128/mBio.00669-17 (PMC5461409; doi:10.1128/mBio.00669-17)
Supplement: FIG S1 [file mbo003173328sf1.pdf]

Supplementary Figure 1

A

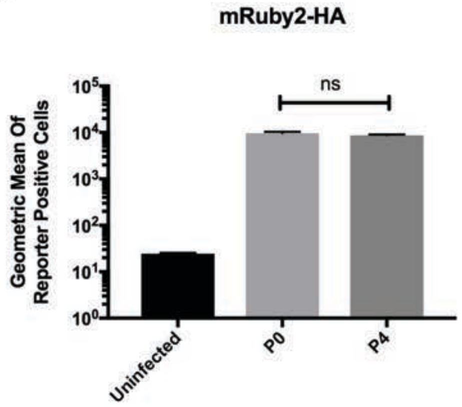

B

mRuby2-HA Passage	Counted Plaques	Positive Plaques
Passage 0	10	10
Passage 4	10	10

C

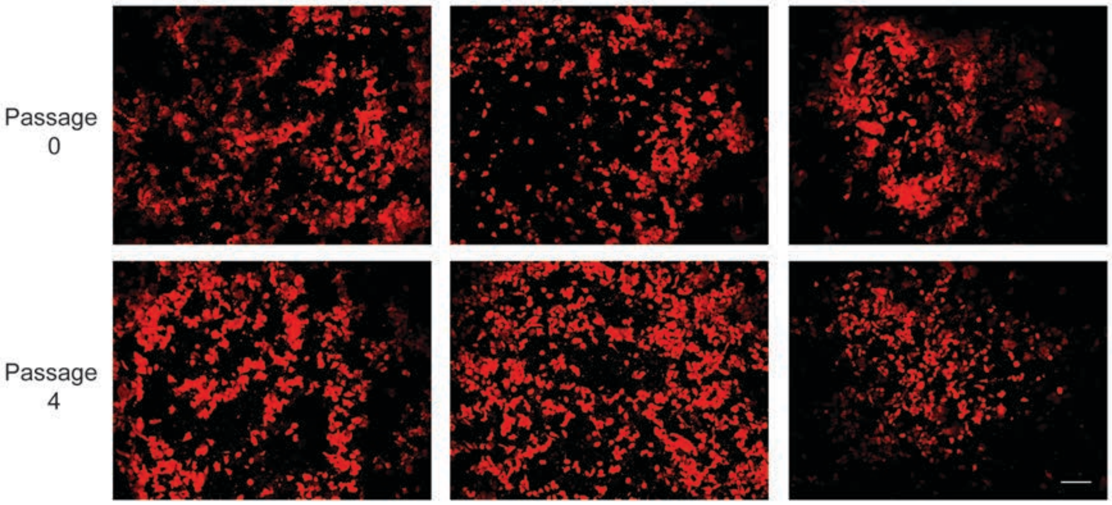

The mRuby2-HA virus stably expresses the reporter protein over serial passaging. (A) Quantification of the brightness of passage 0 and passage 4 mRuby2-HA infected cells via flow cytometry. (**B**) A table comparing the number of reporter positive plaques out of total plaques between passage 0 and passage 4 of the mRuby2-HA virus. Identified plaques were confirmed via staining for flu proteins as described in Methods & Materials. (**C**) Representative images of the plaques that were counted, demonstrating similar brightness and morphology between passages.
