# Supplementary material for: Rationally Designed Influenza Virus Vaccines That Are Antigenically Stable during Growth in Eggs
Source: mBio. 2017 Jun 6;8(3):e00669-17. doi: 10.1128/mBio.00669-17 (PMC5461409; doi:10.1128/mBio.00669-17)
Supplement: FIG S2 [file mbo003173328sf2.pdf]

Supplementary Figure 2

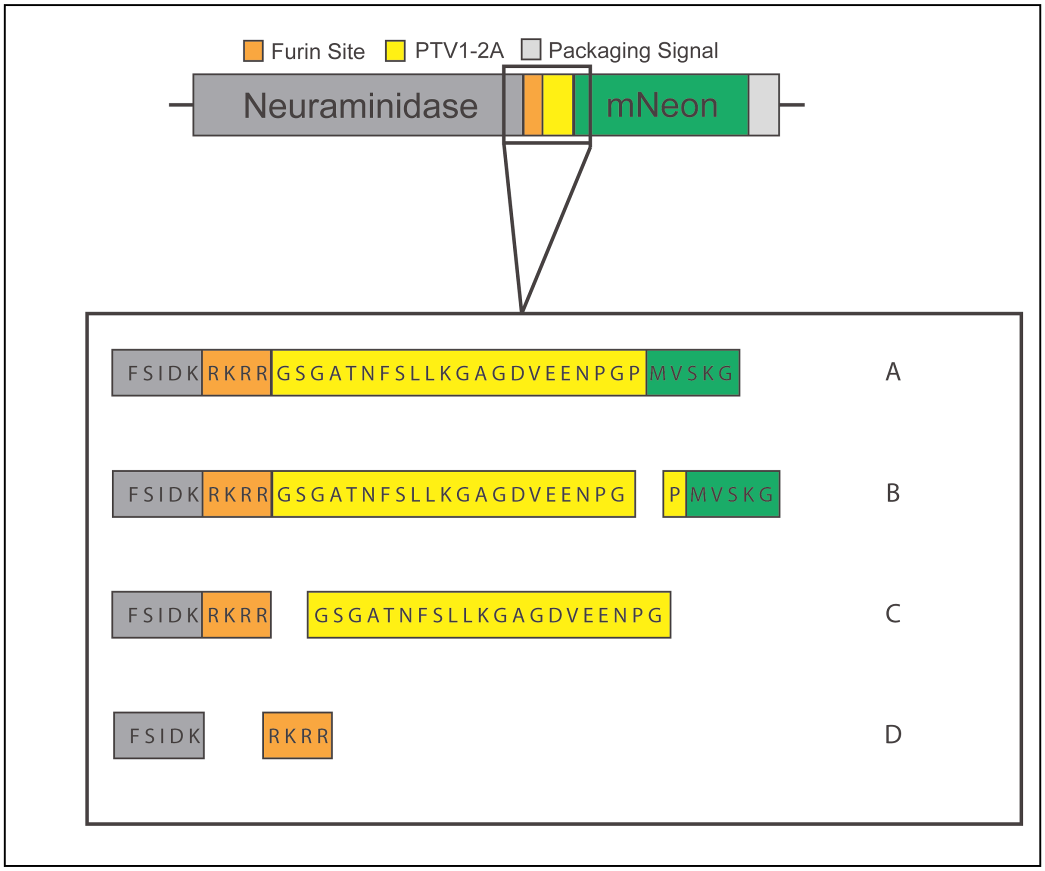

Schematic of the Neuraminidase-Furin-mNeon construct, and it's processing. (**A**) A depiction of the amino acids encoded by the construct, amino acids are color coded to match the specific portions of the construct they come from. (**B**) A depiction of the inability of ribosomes to form a peptide bond between the final Glycine and Proline of the PTV1-2A sequence, causing the Neuraminidase and mNeon proteins to separate. (**C**) A depiction of Furin protease recognizing the cleavage RKRR motif and cleaving the remaining PTV1-2A amino acids from Neuraminidase. (**D**) A depiction of Carboxypetidase B enzymes cleaving the basic amino acids of the furin cleavage site from the N-terminus of Neuraminidase, leaving wild-type protein.
