# Supplementary material for: Rationally Designed Influenza Virus Vaccines That Are Antigenically Stable during Growth in Eggs
Source: mBio. 2017 Jun 6;8(3):e00669-17. doi: 10.1128/mBio.00669-17 (PMC5461409; doi:10.1128/mBio.00669-17)
Supplement: FIG S4 [file mbo003173328sf4.pdf]

Supplementary Figure 4

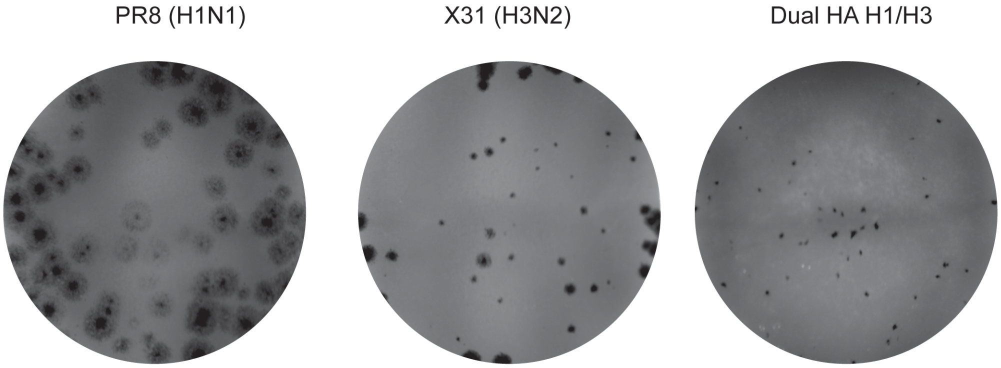

Plaque morphology of the parental PR8 (H1N1), X31 (H3N2) viruses as compared to the Dual HA H1/H3 virus in a plaque assay on MDCK cells.
