# Supplementary material for: Rationally Designed Influenza Virus Vaccines That Are Antigenically Stable during Growth in Eggs
Source: mBio. 2017 Jun 6;8(3):e00669-17. doi: 10.1128/mBio.00669-17 (PMC5461409; doi:10.1128/mBio.00669-17)
Supplement: FIG S5 [file mbo003173328sf5.pdf]

Supplemental Figure 5

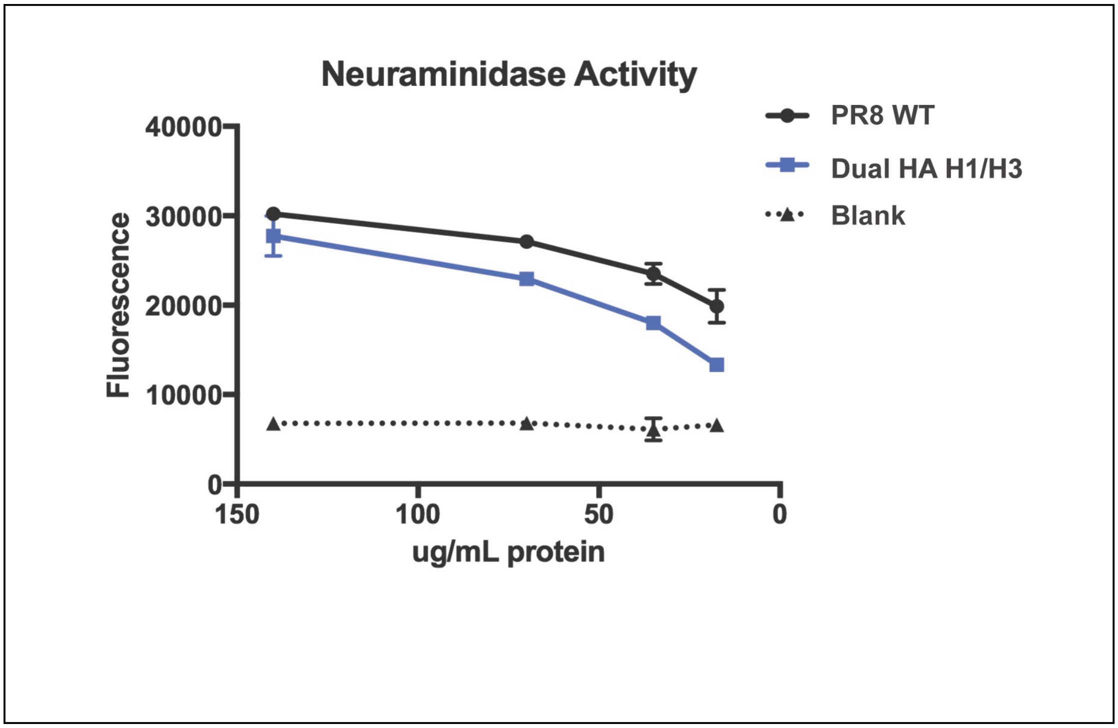

Bivalent viruses have lower neuraminidase activity relative to WT PR8.

Both WT PR8 and the Dual HA H1/H3 virus samples were concentrated and normalized to total protein. A sialidase activity assay was then performed, following the standard procedures of the Sigma-Aldrich Neuraminidase Activity Kit (MAK121), to evaluate NA content of each sample.
