# Supplementary material for: Rationally Designed Influenza Virus Vaccines That Are Antigenically Stable during Growth in Eggs
Source: mBio. 2017 Jun 6;8(3):e00669-17. doi: 10.1128/mBio.00669-17 (PMC5461409; doi:10.1128/mBio.00669-17)
Supplement: TABLE S1 [file mbo003173328st1.docx]

**Supplementary Table 1:**

| Dual HA H1/H3 | Counted Plaques | H3 Positive Plaques | Cumulative Positive Plaques |
| --- | --- | --- | --- |
| Passage A | 6 | 6 | 6/6 |
| Passage B | 7 | 7 | 13/13 |
| Passage C | 7 | 7 | 20/20 |
| Passage D | 4 | 4 | 24/24 |
| Passage E | 5 | 5 | 29/29 |
| Passage F | 5 | 5 | 34/34 |
| Passage G | 3 | 3 | 37/37 |
| Passage H | 5 | 5 | 42/42 |
| Passage I | 3 | 3 | 45/45 |
| Passage J | 2 | 2 | 47/47 |
| Passage K | 3 | 3 | 50/50 |
| Passage L | 3 | 3 | 53/53 |
| Passage M | 5 | 5 | 58/58 |
| Passage N | 5 | 5 | 63/63 |
| Passage O | 5 | 5 | 68/68 |
| Passage P | 5 | 5 | 73/73 |
| Passage Q | 4 | 4 | 77/77 |
| Passage R | 4 | 4 | 81/81 |
| Passage S | 5 | 5 | 86/86 |
| Passage T | 9 | 9 | 95/95 |

**Dual HA A/Hong Kong/1/68-PR8 virus stably expresses second HA in twenty independent parallel passages.** Plaques that stained positive for A/Hong Kong/1/68 HA are shown out of the total plaques counted for each passage.
