# Supplementary material for: Rationally Designed Influenza Virus Vaccines That Are Antigenically Stable during Growth in Eggs
Source: mBio. 2017 Jun 6;8(3):e00669-17. doi: 10.1128/mBio.00669-17 (PMC5461409; doi:10.1128/mBio.00669-17)
Supplement: FIG S6 [file mbo003173328sf6.pdf]

Supplementary Figure 6

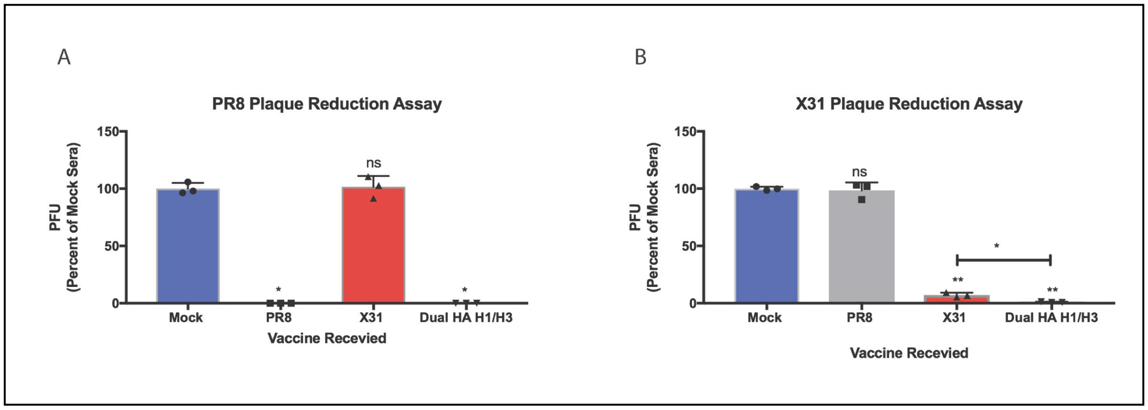

Plaque Reduction Assays performed with sera from vaccinated mice. Plaque reduction assays were repeated at a dilution 2x higher (1:50) than that reported in Figure 5F & G against the PR8 (H1N1) virus (**A**) and X31 (H3N2) virus (**B**).
