# Supplementary material for: Rationally Designed Influenza Virus Vaccines That Are Antigenically Stable during Growth in Eggs
Source: mBio. 2017 Jun 6;8(3):e00669-17. doi: 10.1128/mBio.00669-17 (PMC5461409; doi:10.1128/mBio.00669-17)
Supplement: FIG S7 [file mbo003173328sf7.pdf]

Supplemental Figure 7

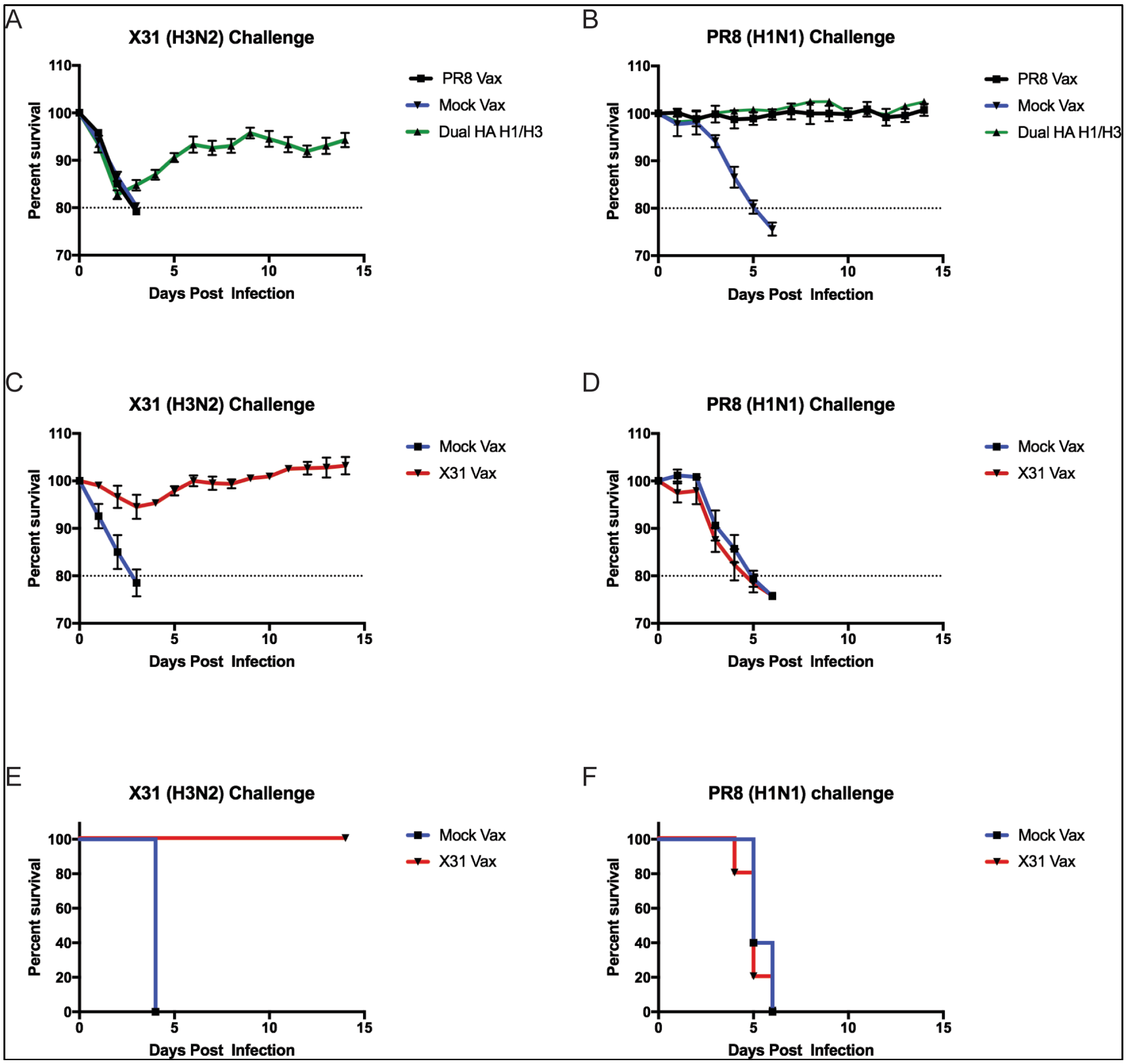

Weight-loss and survival curves from X31 and PR8 challenges of vaccinated mice.

Mice were vaccinated with 7μg of protein from concentrated samples of either inactivated PR8 WT, X31 or Dual HA H1/H3 virus. After 2 weeks mice were boosted and then challenged with the H3N2 strain X31 **(A, C, & E)** or the H1N1 strain PR8 **(B, D, & F)**. Each cage of mice (n $\geq$ 4) was weighed daily for 14 days and the average percent weight-loss was recorded.
