# Supplementary material for: Rationally Designed Influenza Virus Vaccines That Are Antigenically Stable during Growth in Eggs
Source: mBio. 2017 Jun 6;8(3):e00669-17. doi: 10.1128/mBio.00669-17 (PMC5461409; doi:10.1128/mBio.00669-17)
Supplement: FIG S8 [file mbo003173328sf8.pdf]

Supplementary Figure 8

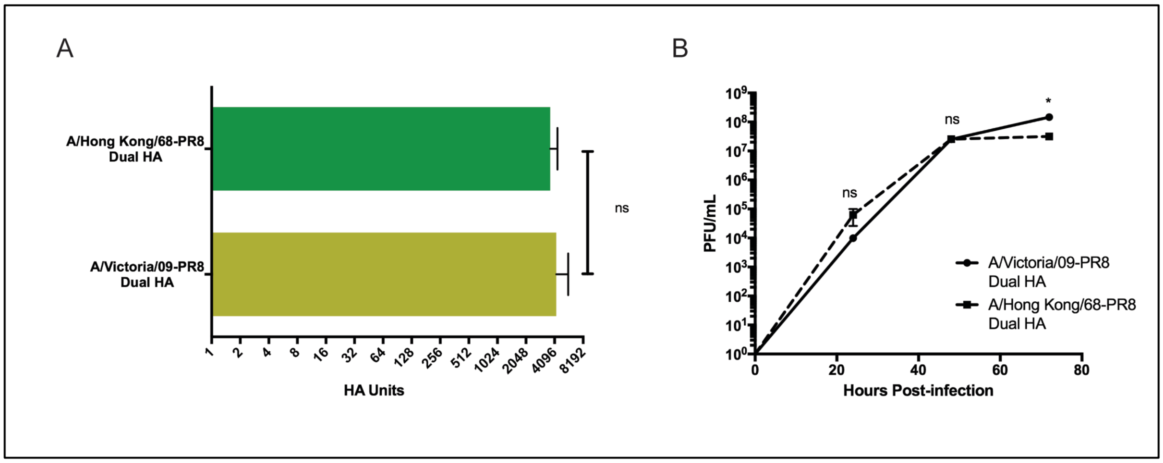

Dual HA viruses using modern H3 HA exhibit similar growth kinetics and HA content to egg-adapted dual HA viruses. (**A**) HA assay of A/Victoria/210/09 expressing dual HA virus as compared to A/Hong Kong/1968 dual HA. (**B**) Growth kinetics in 11-day-old eggs of A/Victoria/210/2009 expressing dual HA virus as compared to the A/Hong Kong/1968 dual HA virus.
