# Supplementary material for: Rationally Designed Influenza Virus Vaccines That Are Antigenically Stable during Growth in Eggs
Source: mBio. 2017 Jun 6;8(3):e00669-17. doi: 10.1128/mBio.00669-17 (PMC5461409; doi:10.1128/mBio.00669-17)
Supplement: TABLE S2 [file mbo003173328st2.docx]

**Supplemental Table 2:**

| **Construct** | **Forward Primer** | **Reverse Primer** |
| --- | --- | --- |
| mRub2-2A-HA Fragment 1 | CTCCGAAGTTGGGGGGGAGCAAAAGCAGG | TTATAGAGTTCATCCATTCCTCCTC |
| mRub2-2A-HA Fragment 2 | ATGGATGAACTCTATAAAGGATCTGGGGCTACCAACTTCAGTCT | TGGGCCGCCGGGTTATTAGTAGAAACAAGG |
| mNeon-2A-HA Fragment 1 | CTCCGAAGTTGGGGGGGAGCAAAAGCAGG | ATATTGTGTCTGCCGCGGCCGCC |
| mNeon-2A-HA Fragment 2 | CGGACGCAGACACAATATGTATAGGCTACCATGCGAACAATTCA | TGGGCCGCCGGGTTATTAGTAGAAACAAGG |
| NA-Furin-2A-mNeon Fragment 1 | CTCCGAAGTTGGGGGGGAGCGAAAGCAGG | CTCCAGTCTACGGTGTCACTATTCACGCCAAAAGAAATGCT |
| NA-Furin-2A-mNeon Fragment 2 | TGACACCGTAGACTGGAGCTGGCCGG | TGGGCCGCCGGGTTATTAGTAGAAACAAGG |
| S4 H1N1 NA/HA Fragment 1 | CTCCGAAGTTGGGGGGGAGCAAAAGCAGG | CCTGCATTCCAAGTGAGAACATGAAA |
| S4 H1N1 NA/HA Fragment 2 | TCTCACTTGGAATGCAGGACCTTTTTTCTGACCCAAGGTGCCTT | GTTGGTAGCCCCAGATGGGGTTCTCTTTCTTTTATCGATAGAAA |
| S4 H1N1 NA/HA Fragment 3 | GGATCTGGGGCTACCAACTTCAGTCT | TGGGCCGCCGGGTTATTAGTAGAAACAAGG |
| NA-Flag-Furin-2A-mNeon Fragment 1 | CTCCGAAGTTGGGGGGGAGCGAAAGCAGG | GTCATCGTCATCTTTATAATCTACCCAGGTGCTATTTTTATAGGTAA |
| NA-Flag-Furin-2A-mNeon Fragment 1 | TATAAAGATGATGATGACAAGGACACAACTTCAGTGATATTAAC | TGGGCCGCCGGGTTATTAGTAGAAACAAGG |
| ZsGreen Seg 6 | CTCCGAAGTTGGGGGGGAGCGAAAGCAGG | TGGGCCGCCGGGTTATTAGTAGAAACAAGG |
| HK 68 HA Seg 6 | CTCCGAAGTTGGGGGGGAGCGAAAGCAGG | TGGGCCGCCGGGTTATTAGTAGAAACAAGG |
| PR8 HA Seg 6 | CTCCGAAGTTGGGGGGGAGCGAAAGCAGG | TGGGCCGCCGGGTTATTAGTAGAAACAAGG |
| Yamagata 88 HA Seg 6 | ACTGGAATTTGCAACCAAGATATCGCCACCATGAAGGCAATAATTGTACTACTCAT | TCTAGCCCTGTTAGCTCAGTTTAAACTTATAGACAGATGGAGCAAGAAACAT |
| Malaysia 04 HA  Seg 6 | ACTGGAATTTGCAACCAAGATATCGCCACCATGAAGGCAATAATTGTACTACTCAT | TCTAGCCCTGTTAGCTCAGTTTAAACTTATAGACAGATGGAGCAAGAAACAT |
| Fujian 2002 HA Seg 6 | ACTGGAATTTGCAACCAAGATATCGCCACCATGAAGACCATCATAGCACTGTCAT | ACAGTCTAGCCCTGTTAGCTCAGTTTAAACTCAGATGCAAATATTACACCGTATA |
| Victoria 2009 HA Seg 6 | ACTGGAATTTGCAACCAAGATATCGCCACCATGAAAACAATTATCGCTC | ACAGTCTAGCCCTGTTAGCTCAGTTTAAACTCAAATACAGATGTTGCAT |

**Primers used in this study – All sequences 5’**
